# Physiopathological Bases of the Disease Caused by *HACE1* Mutations: Alterations in Autophagy, Mitophagy and Oxidative Stress Response

**DOI:** 10.3390/jcm9040913

**Published:** 2020-03-26

**Authors:** Olatz Ugarteburu, Marta Sánchez-Vilés, Julio Ramos, Tamara Barcos-Rodríguez, Gloria Garrabou, Judit García-Villoria, Antonia Ribes, Frederic Tort

**Affiliations:** 1 Section of Inborn Errors of Metabolism-IBC, Department of Biochemistry and Molecular Genetics, Hospital Clínic, IDIBAPS, CIBERER, 08028 Barcelona, Spain; 2 Hospital of Torrecardenas, 04009 Almeria, Spain; 3 Muscle Research and Mitochondrial Function Laboratory, Cellex-IDIBAPS, Faculty of Medicine and Health Science-University of Barcelona, Internal Medicine Service-Hospital Clínic of Barcelona, CIBERER, 08036 Barcelona, Spain

**Keywords:** HACE1, genetic disorder, autophagy, mitophagy, mitochondria, 3-methylglutaconic, oxidative stress

## Abstract

Recessive *HACE1* mutations are associated with a severe neurodevelopmental disorder (OMIM: 616756). However, the physiopathologycal bases of the disease are yet to be completely clarified. Whole-exome sequencing identified homozygous *HACE1* mutations (c.240C>A, p.Cys80Ter) in a patient with brain atrophy, psychomotor retardation and 3-methylglutaconic aciduria, a biomarker of mitochondrial dysfunction. To elucidate the pathomechanisms underlying HACE1 deficiency, a comprehensive molecular analysis was performed in patient fibroblasts. Western Blot demonstrated the deleterious effect of the mutation, as the complete absence of HACE1 protein was observed. Immunofluorescence studies showed an increased number of LC3 puncta together with the normal initiation of the autophagic cascade, indicating a reduction in the autophagic flux. Oxidative stress response was also impaired in HACE1 fibroblasts, as shown by the reduced *NQO1* and *Hmox1* mRNA levels observed in H_2_O_2_-treated cells. High levels of lipid peroxidation, consistent with accumulated oxidative damage, were also detected. Although the patient phenotype could resemble a mitochondrial defect, the analysis of the mitochondrial function showed no major abnormalities. However, an important increase in mitochondrial oxidative stress markers and a strong reduction in the mitophagic flux were observed, suggesting that the recycling of damaged mitochondria might be targeted in HACE1 cells. In summary, we demonstrate for the first time that the impairment of autophagy, mitophagy and oxidative damage response might be involved in the pathogenesis of HACE1 deficiency.

## 1. Introduction

*HACE1* (MIM#610876) encodes for a HECT domain and ankyrin repeat-containing ubiquitin ligase which is reported to be involved in tagging specific target proteins for subcellular localization or for degradation [1]. In addition, this protein is a potential tumor suppressor, as it has been shown to be downregulated in many human tumors [2,3]. In addition, studies performed in cellular and mouse models demonstrated that HACE1 is potentially involved in other physiological processes, including the regulation of the response to oxidative stress [4,5,6] and autophagy [7,8]. 

Recessive mutations in *HACE1* causing loss-of-function have been associated to a severe genetic neurodevelopmental disorder classified as spastic paraplegia and psychomotor retardation with or without seizures (SPPRS) (OMIM: 616756) [9]. Affected individuals presented, in the first months of life or infancy, with a variety of clinical symptoms, including delayed psychomotor development, intellectual disability, epilepsy, hypotonia, spasticity, ataxia, and poor speech skills. Brain abnormalities are also present in some patients and include hypoplastic corpus callosum, cerebral atrophy, delayed myelination and reduced white matter content. Although mutations in *HACE1* have been reported in 18 patients from eight unrelated families, the molecular mechanism underlying the pathogenesis of the disease has not been well determined [10,11,12,13]. In fact, a very recent manuscript reported the unique evidence to date about the pathogenic mechanisms underlying this disorder [13]. This research showed that elevated levels of the Ras-related C3 botulinum toxin substrate 1 protein (RAC1), a well-known target of HACE1, were found in *HACE1*-knockout mice as well as in patient cells, hypothesizing that altered RAC1 signalling might contribute to the pathogenesis of the disorder [13,14]. The role of RAC1 in the physiopathology of HACE1 deficiency is supported by the fact that this protein is involved in brain development and in the regulation of reactive oxygen species (ROS) levels [6,15,16,17]. However, the fact that HACE1 has multiple substrates which participate in different fundamental biological processes suggest that mechanisms other than RAC1 upregulation may also be to blame for this disorder [18].

Here, we present a detailed study that provides further insight into elucidating the potential pathomechanisms underlying HACE1 deficiency. We report a patient carrying *HACE1* mutations presenting with psychomotor retardation, brain atrophy and high urinary excretion of 3-methylglutaconic acid (3-MGA), a well-established biomarker of mitochondrial dysfunction [19]. Molecular studies revealed a severe defect in the regulation of the autophagic flux and a diminished ability to respond to oxidative damage in HACE1 patient cells. The patient phenotype could resemble a mitochondrial defect and the analysis of the mitochondrial function showed altered mitochondrial morphology and a partial disassembly of the oxidative phosphorylation (OXPHOS) system. Notably, an important increase in mitochondrial oxidative stress and a strong reduction in the mitophagic flux were demonstrated, suggesting that the recycling of damaged mitochondria might be targeted in these cells. Altogether, our results demonstrated that *HACE1* mutations lead to a severe neurodevelopmental disorder by targeting key physiological processes such as autophagy, mitophagy and the ability to properly respond to oxidative damage.

## 2. Experimental Section

### 2.1. Case Report

We present the third child of healthy non-consanguineous parents of Pakistani origin. Pregnancy and delivery were uneventful; the birth weight was 3500 g. During the first months of life, the patient presented general psychomotor retardation without signs of regression. At the age of four years, he achieved sedestation, but, currently, at 10 years of age, he is still not able to walk independently. He has severe intellectual disability with greater impairment in the area of language. He displays good connections with people by employing gestures to make him understood, although he only uses familiar names and understands simple orders. Physical examination revealed paresis of the lower extremities with rigidity and exalted osteotendinous reflexes with no other remarkable findings. A brain magnetic resonance imaging (MRI) showed diffuse cortical atrophy and an arachnoid cyst in the right temporal lobe. Routine biochemical analyses were normal, including amino acids and acylcarnitines. The urinary organic acid profile showed persistently elevated levels of 3-metylglutaconic and 3-methylglutaric acids, a biomarker associated to several mitochondrial diseases. 

### 2.2. Whole Exome Sequencing

Trio-exome enrichment and sequencing was performed in the Welcome Trust Sanger Institute. Analysis of the primary data was done in the Centre Nacional d’Anàlisi Genòmica (CNAG) using the RD-Connect Genome-Phenome Analysis Platform (GPAP) standardised analysis pipeline [20]. The variant calls were analyzed using the RD-Connect GPAP (https://platform.rd-connect.eu/).

### 2.3. Cell Culture

Human skin fibroblasts obtained from healthy donors and from a patient with mutations in *HACE1* were maintained in minimum essential medium (MEM) (1 g/L glucose, 10% fetal calf serum and 1% penicillin-streptomycin). Cells were grown to confluence in 25 cm^2^ flasks, harvested by trypsinization and pelleted by centrifugation or reseed.

### 2.4. Protein Expression Analysis

Cell pellets obtained from cultured fibroblasts were homogenized with RIPA buffer (SDS 0.1%, NP40 1%, Sodyum deoxycholate 0.5% in PBS) containing protease inhibitors (cOmplete™, Mini, EDTA-free Protease Inhibitor Cocktail 4693159001, Roche, Indianapolis, IN, USA). Cell lysates were subjected to SDS-PAGE and electroblotted. Proteins were visualized by immunostaining with specific antibodies (Table S1) followed by colorimetric detection (1708235 Opti-4CNTM Substrate Kit, Bio-Rad, Hercules, California, USA). ImageJ software was used for densitometry analysis of protein expression.

Subcellular fractionation was performed in order to obtain total, cytoplasmatic and organelle-enriched fractions. Cells were permeabilized with Lysis Buffer (5 mM Tris/HCl pH 7.4, 250 mM sucrose, 1 mM EDTA, 1 mM EGTA, 1.5 mM MgCl_2_, 1 mM PMSF) containing 0.01% digitonin and centrifuged at 15000× *g* for 10 min at 4 °C. Supernatants (cytoplasmatic fraction) and pellets (membranous fraction) were recovered and subjected to further analysis. 

### 2.5. Blue Native-PAGE Analysis of Mitochondrial Complex and Supercomplex Assembly

Mitochondria-enriched pellets were obtained as described [21]. In order to extract mitochondrial respiratory chain complexes, cells were homogenized with 1% *n*-dodecyl β-D-maltoside. For supercomplex analysis, cells were solubilized with digitonin, a weaker detergent that maintains the interactions between complexes. Lysates were electrophoresed in 4–15% polyacrylamide gradient gels, immunoblotted with specific antibodies against representative subunits of the mitochondrial respiratory chain complexes I–V (Table S1) and visualized by colorimetric detection (1708235 Opti-4CN™ Substrate Kit, Bio-Rad).

### 2.6. High-Resolution Respirometry 

Oxygen consumption of control and HACE1 patient fibroblasts was analyzed by high resolution respirometry using polarographic oxygen sensors in a two-chamber Oxygraph-2k system according to manufacturer’s instructions (Oroboros Instruments, Innsbruck, Austria). Cells were trypsinised and resuspended in MiR05 mitochondrial respiration medium (Oroboros Instruments, Innsbruck, Austria). OXPHOS inhibitors (oligomycin, 04876-5MG, Sigma, St. Louis, MI, USA), antimycin, A8674-25MG, Sigma, St. Louis, MI, USA) and uncoupler carbonyl cyanide 3-chlorophenylhydrazone (CCCP, C2759,Sigma, St. Louis, MI, USA) were manually titrated using Hamilton syringes (Hamilton Company, Reno, NV, USA) as previously described [22]. The concentrations used were 1 μg/mL of oligomycin, stepwise 1 μM titration of CCCP and 2.5 μM of antimycin A. The data were recorded using the DatLab software v7.1.0.21 (Oroboros Instruments, Innsbruck, Austria). 

### 2.7. Mitochondrial Network and Mitochondria Morphology Analysis

The mitochondrial morphology and mitochondrial network were analysed by immunofluorescence followed by confocal microscopy. Cells were grown on glass coverslips, fixed with 4% paraformaldehyde and the reaction was stopped using NH_4_Cl. Fibroblasts were permeabilized with 0.1% TritonX-100 and mitochondria were stained using anti-TOM20 antibody (Table S1), which recognises a mitochondrial membrane protein. Coverslips were mounted with Mowiol 4-88 Mounting Medium (81381 Sigma-Aldrich, Sant Luis, MI, USA) and images were obtained using a Leica TCS SL laser scanning confocal spectral microscope (Leica Microsystems GmbH, Wetzlar, Germany). Analysis was performed using ImageJ software [23]. Mitochondrial length, the ratio between mitochondria length and width (aspect ratio, AR) and the degree of mitochondrial network branching (form factor, FF) were calculated. 

### 2.8. Analysis of Autophagy and Mitophagy in Fibroblasts

We assessed autophagy by analyzing LC3 protein by immunofluorescence and confocal microscopy. Briefly, cells were grown on glass coverslips and fixed with 4% paraformaldehyde and permeabilized using digitonin 100 μg/μL. In order to stain autophagosomes, a primary antibody anti-LC3 (Table S1) was used. After mounting coverslips with Mowiol 4-88 Mounting Medium (81381, Sigma-Aldrich, USA), images were obtained by Leica TCS SL laser scanning confocal spectral microscope (Leica Microsystems GmbH, Wetzlar, Germany). Image analysis was done using ImageJ software and the average number of LC3-II puncta (corresponding to autophagosomal LC-II form) was quantified. In order to analyse the autophagic flux, LC3 puncta was analyzed in the presence and in the absence of 24 h EP (E64D-pepstatin A) treatment, two inhibitors of lysosomal hydrolases (10μM E64D (E8640, Sigma Aldrich, St. Louis, MI, USA) and 10μM Pepstatin A (77170, Sigma Aldrich, St. Louis, MI, USA)). Autophagic flux was determined as the relative increase in LC3-II puncta upon EP treatment compared to those observed at the basal state. 

Mitophagy was assessed by analyzing the colocalization between mitochondrial and LC3 areas, as described [24]. Coverslips were prepared as described above. In this case, we determined the colocalization areas using primary antibodies (Table S1) against LC3, staining autophagosomes, and TOM20, staining the mitochondria. Mitophagy was calculated as the LC3-II/TOM20 colocalization area related to the total mitochondrial area. To monitor the mitophagic flux, the ratio between mitophagy upon EP treatment and without treatment was analysed. We measured the relative increase in LC3-II/TOM20 colocalization areas upon 24 h of EP treatment compared to those detected in the basal state. 

To analyze autophagy induction, cells were subjected to nutrient starvation conditions by exchanging the culture medium for Hank’s Balanced Salt solution (HBSS,14170-088, Gibco, Thermo Fisher Scientific, Waltham, MA, USA) during 2 h. Autophagosome formation was assessed as mentioned above. The nuclear translocation of the transcription factor EB TFEB (Table S1) was determined by immunofluorescence as described [25]. Images were obtained using Eclipse 50 equipment (Nikon instruments Inc., Melville, NY, USA) and the percentage of cells with nuclear TFEB was quantified for each condition.

### 2.9. mRNA Expression Analysis 

Total RNA extraction was performed using QIAshredder and RNeasy kits (74104 Qiagen, Hilden, Germany). We synthesized single-stranded complementary DNA (cDNA) using oligodT primers (C1101 Promega, Madison, WI, USA) and M-MLV Reverse Transcriptase, RNase H Minus, Point Mutant (M368A Promega, Madison, WIS, USA) according to the manufacturer’s protocols. The levels of mRNA expression were analysed by quantitative PCR using specific primers (Table S2). PCR was performed using SYBR Green reagent (4472908, Life Technologies Ltd, Renfrew, UK) in a Step One plus quantitative PCR system (Applied Biosystems, Waltham, MA, USA). In order to study the genes that are upregulated under oxidative stress conditions, cells were treated with H_2_O_2_ (hydrogen peroxide solution H1009, Sigma Aldrich, St. Louis, MI, USA).

### 2.10. Analysis of Mitochondrial Superoxide Levels

Mitochondrial superoxide levels were measured by MitoSOXRed probe (M36008, Invitrogene, Waltham, MA, USA). Fibroblasts were seeded at 80% confluence. Experiments were performed following manufacturer’s indications, incubating cells with 5 µM MitoSOXRed for 10 min at 37 °C. MitoSOXRed containing the medium was removed and cells were rinsed with Hank’s Balanced Salt Solution with calcium and magnesium (HBSS/Ca/Mg, 14025-092, Gibco, Thermo Fisher Scientific, Waltham, MA, USA). Fibroblasts were trypsinised and resuspended in HBSS/Ca/Mg. Fluorescence intensity was analysed by flow cytometry (BD FACS CANTOTM II). Results are shown as the fold increase in the average fluorescence intensity of each sample related to the controls.

### 2.11. Lipid Peroxidation Analysis 

Lipid peroxidation levels were quantified using the BIOXYTECH^®^ LPO-586™ colorimetric assay (Oxys International Inc., Beverlly Hills, CA, USA). Specifically, the levels of malondialdehyde (MDA) and 4-hydroxyalkenal (HAE), which are products of fatty acid peroxide decomposition, were analysed by spectrophotometry. Results are expressed as (μM MDA + HAE)/mg protein.

### 2.12. Statistics

Statistical analyses were performed using the two-tailed Student’s *t* test to compare the means of two independent groups of normally distributed data. The data were reported as the mean ± S.E.E. Values of *p* < 0.05 were considered statistically significant.

## 3. Results

### 3.1. Identification of Mutations in HACE1

The clinical and biochemical phenotype of the patient reported here, in particular the persistently high levels of 3-MGA in urine, set up a strong suspicion of a mitochondrial energy metabolism disorder. However, mutations in all genes previously associated to 3-MGA-uria were ruled out using a self-designed customized gene panel (Haloplex, Agilent Technologies, Santa Clara, CA, USA). In order to elucidate the genetic cause of the disease, we performed whole-exome sequencing of the index case and his healthy parents. Genetic variants were filtered and prioritized as summarized in Figure S1. Data analysis revealed homozygous mutations in *HACE1* (NM_020771.4). The identified mutation (c.240C>A) is predicted to change the cysteine at position 80 of the protein for a translation termination codon (p.Cys80Ter), leading to a potentially truncated protein. Segregation was confirmed by the carrier status of both parents. *HACE1* encodes for a HECT domain and ankyrin repeat-containing E3 ubiquitin ligase 1 protein, involved in tagging specific target proteins for subcellular localization or for degradation [1]. 

### 3.2. HACE1 Protein Expression Is Absent in Patient Fibroblasts 

**Figure 1:**
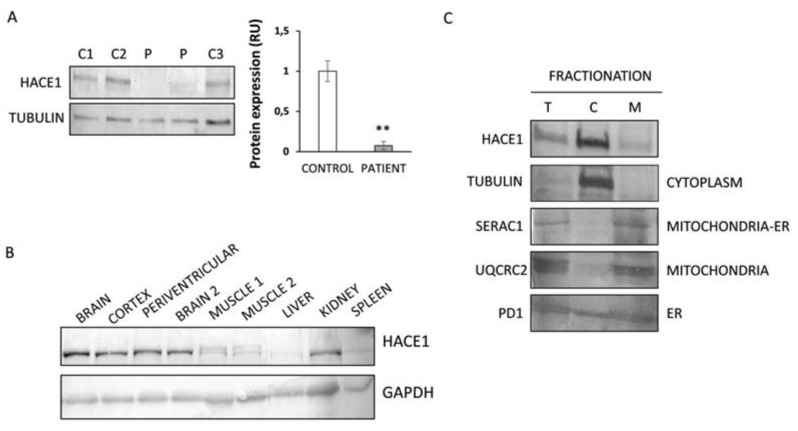
HACE1 protein expression analysis in patient fibroblasts and human tissues. (**A**) Western Blot analysis showed almost absent expression of HACE1 in patient fibroblasts. Extracts from several controls (C1: control 1; C2: control 2 and C3: control 3) and two different extracts from the patient were analysed. Tubulin was used as the loading control. ** *p* < 0.01. (**B**) Protein expression analysis in human control tissues showed a predominant HACE1 expression in brain samples. Glyceraldehyde-3-phosphate dehydrogenase (GAPDH) was used as the loading control. (**C**) Fractionation studies performed in control fibroblasts showed that HACE1 is mainly localized in the cytoplasmic fraction. Protein markers of different organelles (mitochondria, endoplasmic reticulum (ER) and cytoplasm) were studied in order to corroborate proper fractionation. The ER marker (PDI) showed a cytosolic leakage, a phenomenon already observed for this marker [26,27,28]. T: total extract; C: cytoplasmic extract; M: membranous organelle-enriched fraction extract.

To demonstrate the effect of the *HACE1* mutations on the encoded protein, a Western Blot analysis was performed. Results showed almost undetectable levels of HACE1 protein in patient fibroblasts, in contrast to the abundant protein expression levels observed in control individuals (Figure 1A). These results support the predicted disruptive effect of the mutation identified in our patient.

### 3.3. HACE1 Protein Is Mainly Expressed in Brain and Predominantly Localized in the Cytosolic Fraction

To investigate the physiological behavior of HACE1, we studied protein expression in a series of human control tissues (total brain extracts, cortex (grey matter), periventricular (white matter), muscle, heart, kidney, spleen and liver). Results showed that HACE1 was predominantly expressed in brain tissues. Kidneys showed intermediate levels, whereas muscle, liver and spleen showed the lowest levels (Figure 1B). As the patient phenotype was compatible with a mitochondrial disease, we performed a fractionation analysis to determine the subcellular localization of HACE1 in control fibroblasts and evaluate whether HACE1 could be associated to mitochondria. The results demonstrated that HACE1 protein is mainly located in the cytosolic fraction (Figure 1C).

### 3.4. Mitochondrial Function Characterization in HACE1 Patient Fibroblasts

As the HACE1 patient reported here has a biochemical phenotype suggestive of a mitochondrial disorder, we performed an exhaustive characterization of the mitochondrial function.

**Figure 2:**
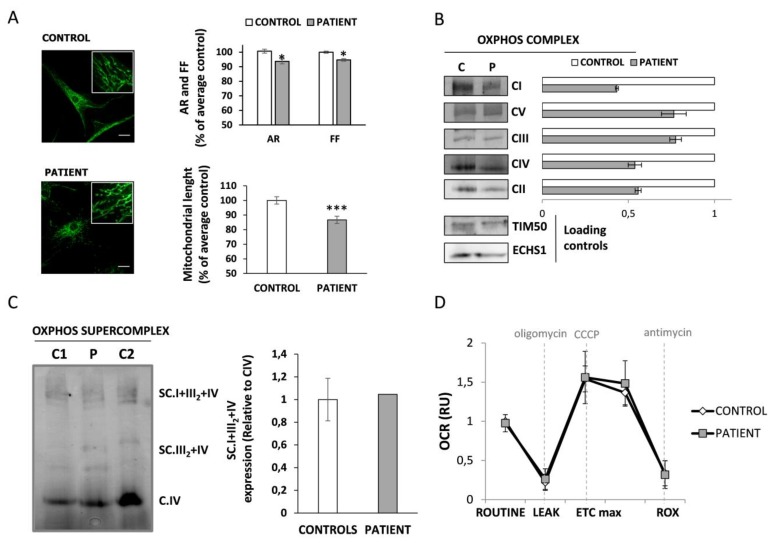
Mitochondrial function in HACE1 fibroblast. (**A**) Mitochondria morphology analysis was performed by immunofluorescence using TOM20 antibody as mitochondrial marker. Results showed a significant reduction in AR (Aspect ratio, indicative of morphology) and FF (Form factor, indicative of mitochondrial network branching) in patient fibroblasts. A significant reduction in the mitochondrial length was also observed in patient cells. A minimum of 70 cells were analysed for each sample. Scale bar, 30 μm. * *p* < 0.05, *** *p* < 0.001. (**B**) Blue Native-PAGE studies and densitometry analysis of OXPHOS complexes showed a partial reduction in the assembly of all complexes in HACE1 patient fibroblasts. The expression of TIM50 (a mitochondrial membrane protein) and ECHS1 (a mitochondrial matrix protein) were used as mitochondrial loading controls. (**C**) Blue Native-PAGE studies followed by densitometry analysis showed no alterations in the assembly of mitochondrial respiratory chain supercomplexes in HACE1 fibroblasts. (**D**) High resolution respirometry analysis showed no differences in the oxygen consumption rate between HACE1 and the control cells. Oxygen consumption rate at basal state (ROUTINE); residual oxygen consumption after oligomycin treatment (LEAK); maximum oxygen consumption induced by carbonyl cyanide 3-chlorophenylhydrazone (CCCP) titration (ETCmax); residual oxygen consumption after antimycin A treatment (ROX). Oxygen consumption rate (OCR) was measured as pmol/(second * millions of cells). The data is expressed as relative units (RU) of control cells.

First, mitochondrial morphology and network branching were analysed by confocal microscopy. Results demonstrated a slight but statistically significant decrease in the parameters aspect ratio (AR) (indicative of morphology) and form factor (FF) (indicative of network branching) in HACE1 cells (*p* < 0.05). In addition, a significant reduction in the mitochondrial length was also observed in patient cells. These results suggest the presence of rounder and shorter mitochondria, as well as reduced network branching degree in HACE1 fibroblasts compared to controls (Figure 2A).

We next assessed the impact of HACE1 deficiency in the OXPHOS system and mitochondrial respiratory capacity. The Blue Native PAGE analysis revealed a generalized decrease in fully assembled individual OXPHOS complexes in HACE1 patient cells (Figure 2B). Similar mitochondrial content was observed in patient and control cells, as demonstrated by the expression levels of TIM50 (mitochondrial membrane protein) and ECHS1 (mitochondrial matrix protein) (Figure 2B, Figure S2). However, these defects had no impact in the assembly of the respirosome, since the levels of OXPHOS supercomplexes were not altered (Figure 2C). We then analysed the mitochondrial respiratory capacity by measuring the oxygen consumption rate (OCR) using high-resolution respirometry. Results showed no significant differences either in the basal respiratory rate or in the maximal respiratory capacity induced upon CCCP stimulation (Figure 2D, Figure S3). Unfortunately, due to material limitation and poor cell growth, a respirometry analysis to determine specific OXPHOS complex deficiencies could not be assessed.

### 3.5. Autophagic Flux Is Altered in HACE1 Patient Fibroblasts

**Figure 3:**
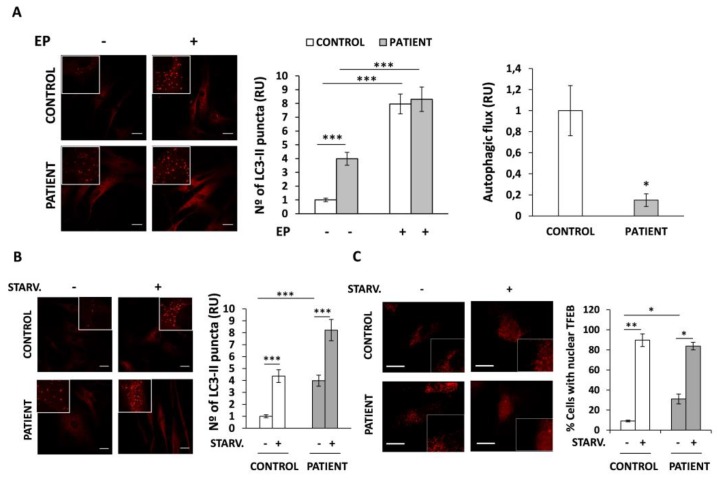
Autophagy analysis in HACE1 patient fibroblasts. Autophagy was measured by immunofluorescence measuring the average number of LC3 puncta per cell and the percentage of cells with TFEB translocated to the nucleus. (**A**) LC3 puncta was significantly increased in HACE1 fibroblasts compared to control cells. The autophagic flux, calculated as the relative increase in LC3puncta after 24 h of EP treatment compared to the basal levels observed in untreated cells, was importantly diminished in HACE1 fibroblasts. (**B**) Upon serum starvation treatment the number of LC3 puncta was increased in both, patient and control fibroblasts, indicating a normal activation of the autophagy. (**C**) The analysis of TFEB by immunofluorescence showed a massive translocation of TFEB to the nucleus after serum starvation treatment in both, control and HACE1 fibroblasts. In all cases a minimum of 70 cells were analysed. Scale bar, 30 μm. Relative units (RU); E64D-Pepstatine A (EP); * *p* < 0.05; ** *p* < 0.01, *** *p* < 0.001.

As HACE1 has been associated with the regulation of autophagy we have analyzed whether this process could be altered in HACE1 patient fibroblasts. Thus, we studied autophagy by immunofluorescence and confocal microscopy by measuring the average number of LC3 puncta per cell, indicative of autophagosomes harboring LC3-II (the lipidated and activated form of LC3). Results revealed that, in the basal state, HACE1 fibroblasts had a marked and significant increase in the number of LC3 puncta (four-fold, *p* < 0.001) compared to control cells (Figure 3A, left panel). 

The fact that autophagy is a highly dynamic process made the rationale for the analysis of the autophagic flux in these cells, in addition to the above-mentioned determination of steady state LC3-II levels. Autophagic flux is defined as the process encompassing autophagosome formation to the delivery and degradation of autophagic substrates into the lysosome. To assess the autophagic flux, LC3 puncta was analyzed in the presence and in the absence of EP treatment, a strong inhibitor of the lysosomal hydrolases. This treatment blocks the degradation of autophagic substrates, including LC3-II, inside the autolysosome. Using this approach, we determined the amounts of LC3 particles which are delivered to lysosomes for degradation during EP treatment. As expected, our results showed that, upon EP treatment, the number of LC3-II puncta was significantly enhanced in both control and patient cells compared to their basal states (*p* < 0.001). Surprisingly, the fold increase in the number of LC3-II particles observed in HACE1 cells was lower (2.1-fold) than in control cells (7.9-fold), regardless of the higher levels observed at the basal state. Therefore, the autophagic flux, defined as the relative increase in LC3-II after EP treatment compared to the basal levels observed in untreated cells, was markedly reduced in HACE1 fibroblasts (Figure 3A, right panel). 

To determine whether the alterations observed in HACE1 fibroblasts could rely on defects in the initial steps of the autophagic cascade, we monitored the formation of LC3-II puncta as well as the nuclear translocation of the transcription factor TFEB in cells treated with nutrient starvation, a well-known autophagy inducer. Results showed a significant increase (*p* < 0.001) in the levels of LC3-II in controls as well as in HACE1 cells upon nutrient deprivation, indicating a rapid induction of autophagy (Figure 3B). In addition, control and HACE1 fibroblasts showed a similar capacity to translocate TFEB to the nucleus, as observed by the massive percentage of cells (89.7% and 83.7%, respectively) harbouring nuclear TFEB in response to serum starvation (Figure 3C). Interestingly, *HACE1*-mutated fibroblasts had an increased percentage of cells with nuclear TFEB at the basal state (31%) in comparison to that observed in controls (9.1%). Altogether, our results suggest that autophagy initiation, at least in response to nutrient deprivation stimuli, may not be significantly affected due to *HACE1* mutations. Moreover, the basal mRNA expression levels of autophagy genes (*LC3*, *p62* and *Beclin1*) in patient cells were similar to those detected in controls (Figure S4).

### 3.6. Mitophagy Is Altered in HACE1 Fibroblasts

**Figure 4:**
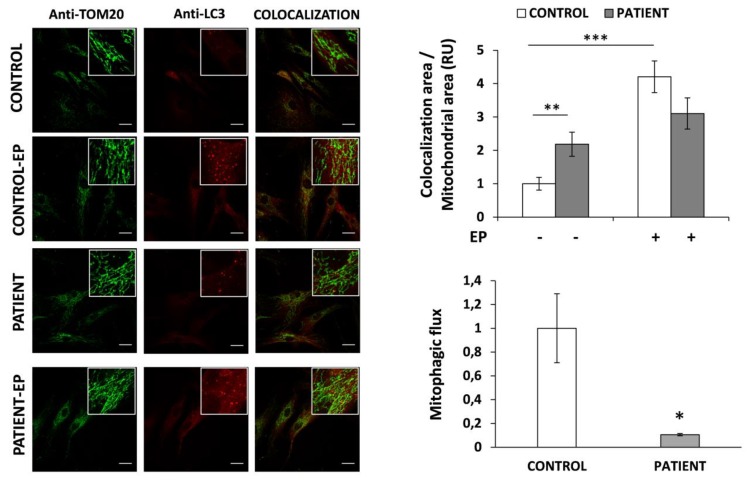
Mitophagy analysis in HACE1 patient fibroblasts. Mitophagv was assessed by immunofluorescence measuring the mitochondria–autophagosome colocalization. Mitochondria was stained with anti-TOM20 and autophagosomes with anti-LC3 antibody. Colocalization was significantly increased in HACE1 fibroblasts compared to control cells. The mitophagic flux, determined as the relative increase in the colocalization area observed after 24 h of EP treatment compared to the basal colocalization, was markedly reduced in HACE1 fibroblasts. In all conditions a minimum of 70 cells were analyzed. Scale bar, 30 μm. Relative units (RU); E64D-Pepstatine A (EP); * *p* < 0.05; ** *p* < 0.01, *** *p* < 0.001.

The suspicion of mitochondrial disorder together with the altered regulation of the autophagic flux detected in patient fibroblasts prompted us to analyze if mitophagy could also be affected in HACE1 cells. Therefore, we evaluated mitophagy by analyzing the colocalization area of the mitochondria (stained with an antibody against TOM20) and autophagosomes (stained with anti-LC3 antibody). Results demonstrated a significantly increased (2.2-fold, *p* < 0.01) mitochondria–autophagosome colocalization area in HACE1 fibroblasts, compared to controls (Figure 4, upper panel). To assess the mitophagic flux we analyzed the colocalization in cells grown with and without EP (an inhibitor of lysosomal degradation of the autophagosomal content). As expected, in control cells, the colocalization area was significantly enhanced upon EP treatment (4.2-fold, *p* < 0.01). In contrast, in HACE1 fibroblasts the effect of EP treatment was milder, resulting in a moderate increase (1.4-fold) of the colocalization respect to the basal situation (Figure 4, upper panel). The mitophagic flux was determined as the relative increase in the mitochondria–autophagosome colocalization area upon EP treatment compared to the basal colocalization. Results demonstrated that, in comparison to controls, the mitophagic flux was markedly reduced in HACE1 fibroblasts (Figure 4, lower panel).

### 3.7. HACE1-Mutated Fibroblasts Accumulate Oxidative Damage and Have a Diminished Ability to Respond to Oxidative Stress 

**Figure 5:**
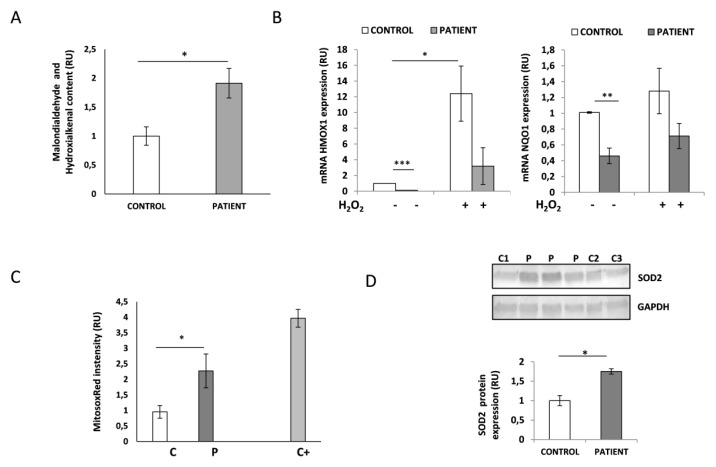
Analysis of oxidative stress in HACE1 patient fibroblasts. (**A**) The quantification of malondialdehyde and hydroxialkenal levels demonstrated higher levels of lipid peroxidation in HACE1 patient fibroblasts. P, patient; C1-C3, controls. * *p* < 0.05. (**B**) Quantitative PCR showed significantly decreased mRNA levels of *HMOX1* and *NQO1* at the basal state as well as upon oxidative stress induced by H_2_O_2_ in HACE1 patient. Experiments were performed in triplicate and results are expressed in relative units (RU). Peptidylprolyl isomerase A (*PPIA*) was used as an internal control. * *p* < 0.05, ** *p* < 0.01, *** *p* < 0.001. (**C**) Mitochondrial superoxide anion levels were analyzed by MitoSOXRed staining and flow cytometry. Increased levels of MitoSOXRed fluorescence intensity were detected in HACE1 patient fibroblasts. Cells treated with antimycin A were used as positive control. Results are expressed in relative units (RU). Patient (P); control (C); positive control (C+); * *p* < 0.05. (**D**) Western Blot analysis demonstrated higher levels of SOD2 protein expression in HACE1 patient fibroblasts. GAPDH was used as the loading control. Cell lysates from several controls (C) and four different extracts from the patient (P) were analyzed. * *p* < 0.05.

Cellular and animal models propose a role for HACE1 in oxidative stress response. To determine whether HACE1 deficiency could lead to oxidative damage, we analyzed lipid peroxidation in the HACE1 patient and control fibroblasts. Lipid peroxidation is one of the consequences derived from the accumulation of high levels of cellular ROS. Therefore, we studied the levels of malondialdehyde and 4-hydroxialkenal content and demonstrated a significant increase (*p* < 0.05) in both products in *HACE1* mutant cells (Figure 5A). These results were indicative of accumulated cellular oxidative damage and raised the question whether the response to oxidative stress could also be impaired in HACE1 fibroblasts and, in consequence, involved in the physiopathology of the disease. To this effect, we analyzed the mRNA expression levels of *NQO1* and *HMOX1*, two well-known genes reported to be upregulated in response to oxidative stress. Interestingly, our results showed that the basal mRNA levels of both genes were significantly decreased in the patient’s fibroblasts (*p* < 0.01). Furthermore, upon H_2_O_2_-induced oxidative stress the induction of *NQO1* and *HMOX1* mRNA expression was also strongly diminished in *HACE1*-mutated cells (Figure 5B).

### 3.8. HACE1 Fibroblasts Had Increased Mitochondrial Oxidative Stress

In order to specifically analyze the presence of mitochondrial oxidative stress, we determined the levels of superoxide anion (the main mitochondrial ROS) in living cells by flow cytometry using a MitoSOXRed probe. Control fibroblasts treated with antimycin, which severely impairs mitochondrial respiratory chain activity and leads to ROS accumulation, were used as a positive control. As expected, antimycin treatment resulted in a significant increase (3.9-fold, *p* < 0.001) in MitoSOXRed intensity compared to untreated cells (Figure 5C). Interestingly, HACE1 fibroblasts showed a significant increase (2.2-fold, *p* < 0.05) in the basal levels of mitochondrial superoxide anion compared to the controls (Figure 5C). In addition, the levels of the mitochondrial antioxidant dismutase enzyme SOD2 analyzed by Western Blot were also increased in HACE1 fibroblasts (*p* < 0.05). Altogether, these results indicate enhanced mitochondrial oxidative stress in *HACE1*-mutated cells (Figure 5D).

## 4. Discussion

In the last decade, the implementation of next generation sequencing technologies has allowed the identification of an increasing number of disease-causing variants in genes associated to a wide variety of cellular processes [29]. However, the physiopathological bases of most rare genetic disorders are yet to be completely elucidated. Here, we report on a patient with psychomotor retardation, brain atrophy and 3-MGA-uria carrying a previously reported homozygous mutation (c.240C>A; p.Cys80Ter) in *HACE1*, which encodes for a HECT domain and ankyrin repeat-containing ubiquitin ligase protein [10]. The identified mutation was predicted to generate a premature termination codon at residue 80; therefore, the disruptive effect was demonstrated by Western Blot, as HACE1 protein expression levels were absent in patient fibroblasts. HACE1 plays an important role in tagging specific proteins for subcellular localization or proteasomal degradation [1] and, as confirmed here by Western Blot analysis, it is mainly expressed in brain tissues [13,30,31].

To date *HACE1* bi-allelic mutations have been reported in eight unrelated families [10,11,12,13]. The clinical data of the previously described individuals do not differ from the phenotype of the patient reported here, but 3-MGA-uria has not been reported in any of the previous cases. Although the true origin of 3-MGA excretion is not well known, the presence of this metabolite in urine is considered a good biomarker of mitochondrial dysfunction; in particular, it has been associated to mutations in genes encoding for proteins related to the mitochondrial membrane [19,32]. Even though the patient phenotype could resemble a mitochondrial disorder, a subcellular fractionation analysis performed in control fibroblasts demonstrated that HACE1 mainly localizes in the cytosolic fraction. These results are concordant with previous observations made in NIH3T3 murine cells, which showed that HACE1 was barely expressed in the mitochondria but cofractionate with cytosolic and endoplasmatic reticulum markers [30]. Since 3-MGA-uria is frequently associated to alterations of the mitochondrial morphology and the assembly of the OXPHOS system [19,32], we performed a detailed analysis of the mitochondrial function in *HACE1* mutated cells. The results showed alterations in the mitochondrial morphology and a partial disassembly of the OXPHOS system. However, these alterations have no impact on the formation of the mitochondrial respiratory supercomplexes or in the cellular respiratory capacity.

Although mutations in *HACE1* have already been reported, the physiopathological mechanisms underlying this disorder are not yet fully understood. To this regard, a very recent publication provided the first and, so far, most unique insight into the molecular consequences of HACE1 deficiency in human disease [13]. The authors demonstrated that RAC1, a well-known HACE1 target associated to brain development and regulation of intracellular ROS, is importantly upregulated in HACE1 patient cells. Indeed, ROS levels were also increased in these cells, pointing that altered RAC1 signalling might contribute to the pathogenesis of the disorder. However, it has been postulated that RAC1 upregulation would not explain the whole phenotypic spectrum of this disease, suggesting that other molecular pathways might also be involved [18]. Interestingly, several studies performed in different organisms and cellular models have implicated HACE1 in the regulation of other fundamental cellular processes, including autophagy [7,8], activation of antioxidant response and cell survival upon oxidative damage [4,5]. In the present study, we wondered whether these pathways could also be impaired in HACE1 patient fibroblasts and contribute to the physiopathology of the disease.

Autophagy is an evolutionarily conserved process that recycles cellular components and organelles by targeting intracytoplasmic cargo to lysosomes for degradation [33,34,35,36]. In addition to lysosomes, the proper function of this process requires the formation of autophagosomes, highly specialized double-membraned organelles that enclose a portion of cytoplasmatic material for further fusion and delivery to lysosomes. In the present study, we have analyzed autophagy by evaluating LC3 protein. LC3 is localized in the cytosol (LC3-I form) until it becomes activated and re-localized to the autophagosome membranes (LC3-II form). Autophagosomes can be detected and quantified by immunofluorescence as bright LC3 positive puncta [37]. Using this approach, we demonstrated a pronounced increase in LC3 puncta in patient fibroblasts when compared to control cells. These results indicate that, in the basal state, autophagosomes are abnormally accumulated in *HACE1* mutated cells. However, since autophagy is a dynamic process, the accumulation of LC3 particles can be promoted either by autophagy activation or by a blockade of downstream stages. In fact, the basal number of autophagosomes is the balance between the rate of autophagosome formation and the rate of autophagosome fusion with the lysosomes and the subsequent degradation of their content [34]. Interestingly, the mRNA levels of *LC3* as well as other autophagy regulators, such as *p62* and *Beclin 1,* were not increased in *HACE1* mutated cells. These observations suggested that the higher number of LC3 puncta observed in patient fibroblasts might be due to post transcriptional regulatory mechanisms rather than an induction of autophagy via the activation of mRNA transcription. Moreover, autophagy initiation seemed to be unaltered in HACE1-deficient cells since nutrient deprivation treatment, a well-known and potent autophagy inducer, provoked a massive nuclear translocation of TFEB and a rapid generation of autophagomes in both patient and control fibroblasts [25,34]. We next measured the autophagic flux by analyzing the LC3-II turnover, treating the cells with EP, a strong inhibitor of the lysosomal hydrolases that allows autolysosome formation but blocks the degradation of the autolysosomal content [38]. We demonstrated that the autophagic flux, calculated as the relative increase in LC3-II levels observed upon EP treatment compared to those in the basal state, was strongly decreased in the HACE1 patient’s fibroblasts. Our results are consistent with the reported observations that showed an abnormal accumulation of LC3 protein in *HACE1*-knockout mice and also demonstrate that HACE1 is involved in the late stages of the autophagosome maturation process [7]. These observations, along with the new data reported here, suggest that the reduced autophagic flux detected in the HACE1 patient might be due to delayed completion of the autophagy pathway. Our results provide the first evidence of impaired autophagy in HACE1 patients and propose the involvement of this mechanism in the physiopathology of the disease.

Several studies performed in mice and cell models proposed a role for HACE1 in the regulation of the oxidative stress response [4,5,6]. The principal consequence of a poor management of cellular redox disturbances is the increase in ROS levels, which react with and damage cellular components such as proteins, lipids and DNA [39,40,41]. Here, we demonstrated an abnormal accumulation of ROS-derived cellular damage in *HACE1* mutated fibroblasts, as seen by increased lipid peroxidation. In addition to RAC1 regulation, HACE1 is also involved in ROS detoxification by promoting the stability of the nuclear factor erythroid 2-related factor 2 (NRF2), a master regulator of the antioxidative response [4,5,42]. Under oxidative stress conditions NRF2 transcription factor translocates to the nucleus and induces the expression of a plethora of genes involved in the response to oxidative damage [43,44,45]. Indeed, it has been shown that, upon oxidative stress, the proper induction of NRF2 target genes is severely impaired in cells derived from *HACE1* knockout mice [4,5]. Notably, we demonstrated that in HACE1 patient fibroblasts, the mRNA levels of *NQO1* and *HMOX1,* two NRF2 transcriptional targets, reported to be upregulated in response to oxidative stress, were significantly reduced even upon H_2_O_2_ treatment. These results suggest that the accumulation of oxidative damage in HACE1 fibroblasts could rely, at least in part, on the diminished ability of these cells to respond to oxidative stress via NRF2. Our data provide further evidence supporting the role of oxidative damage in the pathogenesis of HACE1 deficiency and complement the recently reported observations made by Nagy et al. (2019) [13].

As discussed above, the phenotype of the HACE1 patient reported here resembles a mitochondrial disorder. Mitochondria are the major source of intracellular ROS, where incomplete reduction of oxygen or electron leakage from the mitochondrial respiratory chain generates superoxide radical anions [39,40,41]. The fact that oxidative damage is importantly accumulated in HACE1 fibroblasts prompted us to specifically analyze mitochondrial ROS in these cells. Increased levels of superoxide radical anions and high SOD2 expression evidenced that mitochondrial oxidative stress was also present in *HACE1* mutated cells. These observations could be controversial since it has been reported that SOD2 is also a target of NRF2. Nevertheless, the oxidative damage response pathways are complex and involve a large number of proteins and transcription factors. Therefore, the high levels of SOD2 detected in HACE1-deficient cells could be due to the action of factors other than NRF2, such as PGC-1α, a master co-regulator of energy metabolism, SIRT3, among others [40]. These observations together with the persistently high levels of 3-MGA-uria and slight mitochondrial morphology alterations made the rationale to determine whether mitophagy, the selective form of autophagy that recycles damaged mitochondria, could also be targeted [46]. Mitophagy was determined by immunofluorescence, measuring the mitochondria–autophagosome colocalization area [24]. Similar to the results observed for general autophagy, the mitophagic flux was strongly decreased in patient cells, concluding that the turnover of damaged mitochondrial was also impaired as a consequence of *HACE1* mutations. However, future experiments using more sophisticated analysis methods [47,48] will be required in order to obtain quantitative mitophagy data and to improve the knowledge of the defects caused by HACE1 deficiency.

## 5. Conclusions

In summary, the present study, together with the recently reported observations of Nagy et al. (2019) [13], represents the first characterization of the molecular mechanisms underlying HACE1 deficiency in human disease. Here, we demonstrated that *HACE1* mutations lead to a severe impairment of the autophagic flux and a decreased ability to respond to oxidative damage. In addition, we also showed increased mitochondrial oxidative stress and altered mitophagy in HACE1 patient cells. Altogether, our observations suggest, for the first time, that these physiological processes may be involved in the pathogenesis of this disorder. However, future studies based on functional complementation of HACE1 patients’ fibroblasts and the analysis of HACE1-deficient neuronal cell lines will be required to gain further knowledge about the contribution of altered autophagy, mitophagy and oxidative stress impairment in the physiopathology of this disorder.

## Supplementary Materials

The following are available online at https://www.mdpi.com/2077-0383/9/4/913/s1, Figure S1: Identification of *HACE1* mutations. Figure S2: Quantification of TIM50 and ECHS1 protein expression. Figure S3: High resolution respirometry analysis normalized to protein content. Figure S4. mRNA expression analysis of autophagy genes. Table S1: Antibodies used in this study. Table S2: Oligonucleotides used in this study.

## References

[1] Scheffner M., Kumar S. (2014). Mammalian HECT ubiquitin-protein ligases: Biological and pathophysiological aspects. Biochim. Biophys. Acta-Mol. Cell Res..

[2] Fernandez C.V., Lestou V.S., Wildish J., Lee C.L.Y., Sorensen P.H.B. (2001). Detection of a novel t (6;15) (q21;q21) in a pediatric Wilms tumor. Cancer Genet. Cytogenet..

[3] Zhang L., Anglesio M.S., O’Sullivan M., Zhang F., Yang G., Sarao R., Nghiem M.P., Cronin S., Hara H., Melnyk N. (2007). The E3 ligase HACE1 is a critical chromosome 6q21 tumor suppressor involved in multiple cancers. Nat. Med..

[4] Rotblat B., Southwell A.L., Ehrnhoefer D.E., Skotte N.H., Metzler M., Franciosi S., Leprivier G., Somasekharan S.P., Barokas A., Deng Y. (2014). HACE1 reduces oxidative stress and mutant Huntingtin toxicity by promoting the NRF2 response. Proc. Natl. Acad. Sci. USA.

[5] Ehrnhoefer D.E., Southwell A.L., Sivasubramanian M., Qiu X., Villanueva E.B., Xie Y., Waltl S., Anderson L., Fazeli A., Casal L. (2018). HACE1 is essential for astrocyte mitochondrial function and influences Huntington disease phenotypes in vivo. Hum. Mol. Genet..

[6] Daugaard M., Nitsch R., Razaghi B., McDonald L., Jarrar A., Torrino S., Castillo-Lluva S., Rotblat B., Li L., Malliri A. (2013). Hace1 controls ROS generation of vertebrate Rac1-dependent NADPH oxidase complexes. Nat. Commun..

[7] Zhang L., Chen X., Sharma P., Moon M., Sheftel A.D., Dawood F., Nghiem M.P., Wu J., Li R.K., Gramolini A.O. (2014). HACE1-dependent protein degradation provides cardiac protection in response to haemodynamic stress. Nat. Commun..

[8] Liu Z., Chen P., Gao H., Gu Y., Yang J., Peng H., Xu X., Wang H., Yang M., Liu X. (2014). Ubiquitylation of Autophagy Receptor Optineurin by HACE1 Activates Selective Autophagy for Tumor Suppression. Cancer Cell.

[9] OMIM Online Mendelian Inheritance in Man, OMIM^®^. McKusick-Nathans Institute of Genetic Medicine, Johns Hopkins University (Baltimore, MD). http://omim.org/.

[10] Akawi N., McRae J., Ansari M., Balasubramanian M., Blyth M., Brady A.F., Clayton S., Cole T., Deshpande C., Fitzgerald T.W. (2015). Discovery of four recessive developmental disorders using probabilistic genotype and phenotype matching among 4125 families. Nat. Genet..

[11] Hollstein R., Parry D.A., Nalbach L., Logan C.V., Strom T.M., Hartill V.L., Carr I.M., Korenke G.C., Uppal S., Ahmed M. (2015). HACE1 deficiency causes an autosomal recessive neurodevelopmental syndrome. J. Med. Genet..

[12] Hariharan N., Ravi S., Pradeep B.E., Subramanyam K.N., Choudhary B., Srinivasan S., Khanchandani P. (2018). A novel loss-of-function mutation in HACE1 is linked to a genetic disorder in a patient from India. Hum. Genome Var..

[13] Nagy V., Hollstein R., Pai T.P., Herde M.K., Buphamalai P., Moeseneder P., Lenartowicz E., Kavirayani A., Korenke G.C., Kozieradzki I. (2019). HACE1 deficiency leads to structural and functional neurodevelopmental defects. Neurol. Genet..

[14] Torrino S., Visvikis O., Doye A., Boyer L., Stefani C., Munro P., Bertoglio J., Gacon G., Mettouchi A., Lemichez E. (2011). The E3 ubiquitin-ligase HACE1 catalyzes the ubiquitylation of active Rac1. Dev. Cell.

[15] Luo L., Hensch T.K., Ackerman L., Barbel S., Jan L.Y., Jan Y.N. (1996). Differential effects of the Rac GTPase on Purkinje cell axons and dendritic trunks and spines. Nature.

[16] Stankiewicz T.R., Linseman D.A. (2014). Rho family GTPases: Key players in neuronal development, neuronal survival, and neurodegeneration. Front. Cell. Neurosci..

[17] Mulherkar S., Uddin M.D., Couvillon A.D., Sillitoe R.V., Tolias K.F. (2014). The small GTPases RhoA and Rac1 regulate cerebellar development by controlling cell morphogenesis, migration and foliation. Dev. Biol..

[18] Deng H.X. (2019). HACE1, RAC1, and what else in the pathogenesis of SPPRS?. Neurol. Genet..

[19] Wortmann S.B., Kluijtmans L.A., Engelke U.F.H., Wevers R.A., Morava E. (2012). The 3-methylglutaconic acidurias: What’s new?. J. Inherit. Metab. Dis..

[20] Laurie S., Fernandez-Callejo M., Marco-Sola S., Trotta J.R., Camps J., Chacón A., Espinosa A., Gut M., Gut I., Heath S. (2016). From Wet-Lab to Variations: Concordance and Speed of Bioinformatics Pipelines for Whole Genome and Whole Exome Sequencing. Hum. Mutat..

[21] Wittig I., Braun H.P., Schägger H. (2006). Blue native PAGE. Nat. Protoc..

[22] Pesta D., Gnaiger E. (2012). High-Resolution Respirometry: OXPHOS Protocols for Human Cells and Permeabilized Fibers from Small Biopsies of Human Muscle. Methods Mol. Biol..

[23] Schindelin J., Arganda-Carreras I., Frise E., Kayning V., Longair M., Pietzsch T., Preibisch S., Rueden C., Saalfeld S., Schmid B. (2012). Fiji: An open-source platform for biological-image analysis. Nat. Methods.

[24] Diot A., Hinks-Roberts A., Lodge T., Liao C., Dombi E., Morten K., Brady S., Fratter C., Carver J., Muir R. (2015). A novel quantitative assay of mitophagy: Combining high content fluorescence microscopy and mitochondrial DNA load to quantify mitophagy and identify novel pharmacological tools against pathogenic heteroplasmic mtDNA. Pharmacol. Res..

[25] Medina D.L., Settembre C., Ballabio A., Galluzi L., Bravo-San Pedro J.M., Kroemer G. (2017). Methods to Monitor and Manipulate TFEB Activity During Autophagy. Methods in Enzymology. Molecular Characterization of Autophagic Responses Part, B.

[26] Kumar S., Flacke J.P., Kostin S., Appukuttan A., Reusch H.P., Ladilov Y. (2011). SLC4A7 sodium bicarbonate co-transporter controls mitochondrial apoptosis in ischaemic coronary endothelial cells. Cardiovasc. Res..

[27] Zehmer J.K., Bartz R., Liu P., Anderson R.G.W. (2008). Identification of a novel N-terminal hydrophobic sequence that targets proteins to lipid droplets. J. Cell Sci..

[28] Terada K., Manchikalapudi P., Noiva R., Jauregui H.O., Stockert R.J., Schilsky M.L. (1995). Secretion, surface localization, turnover, and steady state expression of protein disulfide isomerase in rat hepatocytes. J. Biol. Chem..

[29] Boycott K.M., Hartley T., Biesecker L.G., Gibbs R.A., Innes A.M., Riess O., Belmont J., Dunwoodie S.L., Jojic N., Lassmann T. (2019). A Diagnosis for All Rare Genetic Diseases: The Horizon and the Next Frontiers. Cell.

[30] Anglesio M.S., Evdokimova V., Melnyk N., Zhang L., Fernandez C.V., Grundy P.E., Leach S., Marra M.A., Brooks-Wilson A.R., Penninger J. (2004). Differential expression of a novel ankyrin containing E3 ubiquitin-protein ligase, Hace1, in sporadic Wilms’ tumor versus normal kidney. Hum. Mol. Genet..

[31] Nagase T., Kikuno R., Ishikawa K.I., Hirosawa M., Ohara O. (2000). Prediction of the coding sequences of unidentified human genes. XVI. The complete sequences of 150 new cDNA clones from brain which code for large proteins in vitro. DNA Res..

[32] Jones D.E., Perez L., Ryan R.O. (2020). 3-Methylglutaric acid in energy metabolism. Clin. Chim. Acta.

[33] Levine B., Kroemer G. (2008). Autophagy in the Pathogenesis of Disease. Cell.

[34] Mizushima N., Yoshimori T., Levine B. (2010). Methods in Mammalian Autophagy Research. Cell.

[35] Rubistein D.C. (2006). The roles of intracellular protein-degradation pathways in neurodegeneration. Nature.

[36] Peker N., Gozuacik D. Autophagy as a cellular stress response mechanism in the nervous system. J. Mol. Biol..

[37] Kabeya Y., Mizushima N., Ueno T., Yamamoto A., Kirisako T., Noda T., Kominami E., Ohsumi Y., Yoshimori T. (2000). LC3, a mammalian homologue of yeast Apg, is localized in autophagosomemembranes after processing. EMBO J..

[38] Tanida I., Minematsu-Ikeguchi N., Ueno T., Kominami E. (2005). Lysosomal turnover, but not a cellular level, of endogenous LC3 is a marker for autophagy. Autophagy.

[39] Zorov D.B., Juhaszova M., Sollott S.J. (2014). Mitochondrial reactive oxygen species (ROS) and ROS-induced ROS release. Physiol. Rev..

[40] Olsen R.K.J., Cornelius N., Gregersen N. (2015). Redox signalling and mitochondrial stress responses; lessons from inborn errors of metabolism. J. Inherit. Metab. Dis..

[41] Hamanaka R.B., Chandel N.S. (2010). Mitochondrial reactive oxygen species regulate cellular signaling and dictate biological outcomes. Trends Biochem. Sci..

[42] Malhotra D., Portales-Casamar E., Singh A., Srivastava S., Arenillas D., Happel C., Shyr C., Wakabayashi N., Kensler T.W., Wasserman W.W. (2010). Global mapping of binding sites for Nrf2 identifies novel targets in cell survival response through chip-seq profiling and network analysis. Nucleic Acids Res..

[43] Kensler T.W., Wakabayashi N., Biswal S. (2007). Cell survival responses to environmental stresses via the Keap1-Nrf2-ARE pathway. Annu. Rev. Pharmacol. Toxicol..

[44] Wild A.C., Moinova H.R., Mulcahy R.T. (1999). Regulation of gamma-glutamylcysteine synthetase subunit gene expression by the transcription factor Nrf2. J. Biol. Chem..

[45] Itoh K., Chiba T., Takahashi S., Ishii T., Igarashi K., Katoh Y., Oyake T., Hayashi N., Satoh K., Hatayama I. (1997). An Nrf2/small Maf heterodimer mediates the induction of phase II detoxifying enzyme genes through antioxidant response elements. Biochem. Biophys. Res. Commun..

[46] Youle R.J., Narenda D.P. (2011). Mechanisms of mitophagy. Nat. Rev. Mol. Cell. Biol..

[47] McWilliams T.G., Ganley I.G. (2016). Life in lights: Tracking mitochondrial delivery to lysosomes in vivo. Autophagy.

[48] McWilliams T.G., Prescott A.R., Allen G.F., Tamjar J., Munson M.J., Thomson C., Muqit M.M., Ganley I.G. (2016). mito-QC illuminates mitophagy and mitochondrial architecture in vivo. J. Cell Biol..

