# Supplementary material for: Physiopathological Bases of the Disease Caused by HACE1 Mutations: Alterations in Autophagy, Mitophagy and Oxidative Stress Response
Source: J Clin Med. 2020 Mar 26;9(4):913. doi: 10.3390/jcm9040913 (PMC7231286; doi:10.3390/jcm9040913)
Supplement: Supplementary file 1 [file jcm-09-00913-s001.pdf]

Supplementary Material

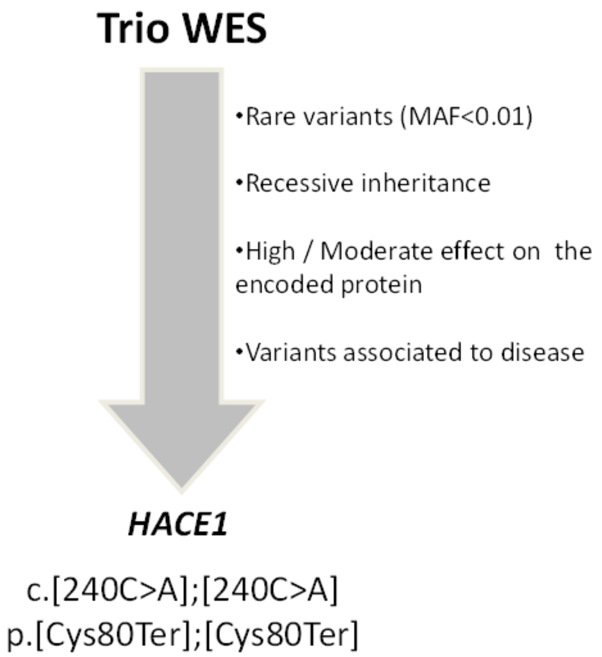

Figure S1. Identification of *HACE1* mutations. Exome data analysis and filtering steps leading to the identification of mutations in *HACE1*. MAF, minor allele frequency.

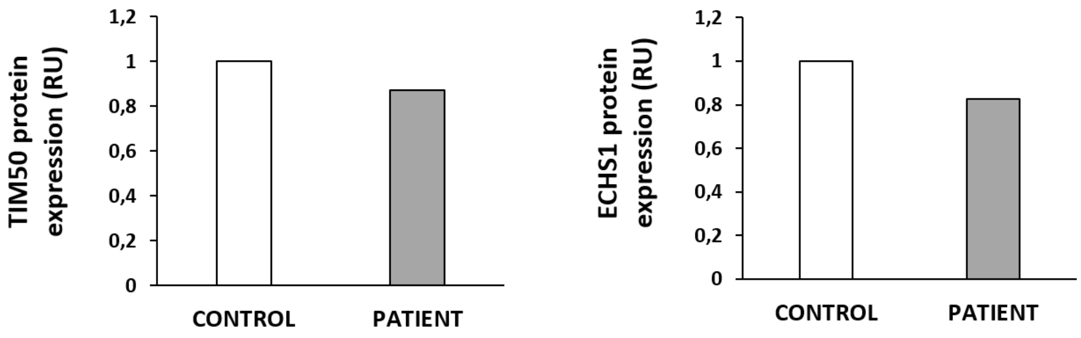

Figure S2. Quantification of TIM50 and ECHS1 protein expression. Western Blot analysis of TIM50 (mitochondrial membrane protein) and ECHS1 (mitochondrial matrix protein) showed similar expression levels between control and patient cells, indicating no differences in mitochondrial content. Results are expressed in relative units (RU).

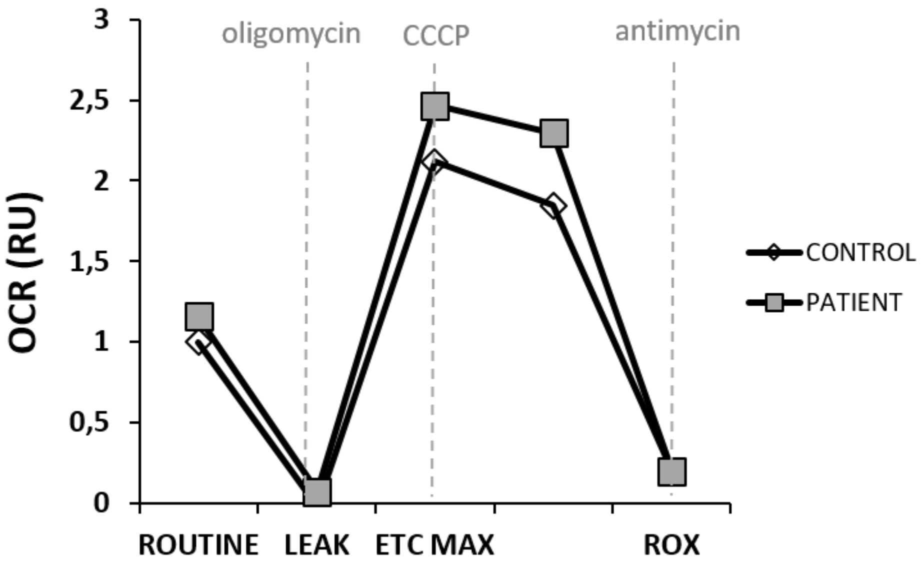

Figure S3. High resolution respirometry analysis normalized to protein content. High resolution respirometry analysis normalized to protein content showed no major differences in the oxygen consumption rate (OCR) between HACE1 and control cells. ROUTINE, oxygen consumption rate at basal state; LEAK, residual oxygen consumption after oligomycin treatment; ETCmax, maximum oxygen consumption induced by CCCP titration; ROX, residual oxygen consumption after antimycin A treatment. OCR was normalized to protein content. Data is expressed as relative units (RU) of control cells.

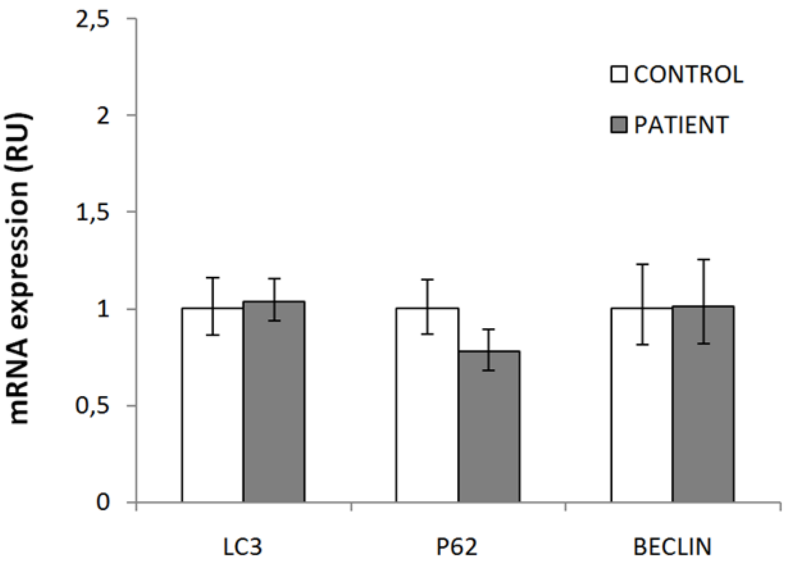

Figure S4. mRNA expression analysis of autophagy genes. Quantitative PCR showed significantly similar mRNA expression levels in HACE1 patient and control fibroblasts. Experiments were performed in triplicate and results are expressed in relative units (RU). PPIA was used as internal control.

Table S1. Antibodies used in this study

Protein	Reference	Manufacturer
NDUFA9	459100	ThermoScientific, USA
SDHA	MS204	MitoSciences, USA
UQCRC2	ab14745	Abcam, UK
UQCRFS1	ab14746	Abcam, UK
COX5A	ab110262	Abcam, UK
ATP5A	ab14748	Abcam, UK
HACE1	ab133637	Abcam, UK
PDI	MA3-019	ThermoScientific, USA
SERAC1	HPA025716	Sigma Aldrich, USA
SOD2	HPA001814	Sigma Aldrich, USA
LC3	PM036	MBL, USA
TOM20	Sc-11415	Sigma Aldrich, USA
α –TUBULIN	ab176560	Abcam, UK
GAPDH	sc-47724	Santa Cruz Biotechnology, USA

Table S2. Oligonucleotides used in this study.

Gene	Forward	Reverse
NQO1	5′-GCCGCAGACCTTGTGATATT-3′	5′-CTGGTTTGAGCGAGTGTTCA-3′
HMOX1	5′-AACTTTCAGAAGGGCCAGGT	5′GTAGACAGGGGCGAAGACTG-3′
LC3	5′CATGAGCGAGTTGGTCAAGA	5′CTCGTCTTTCTCCTGCTCGT-3′
SQSTM1	5′GCACCCCAATGTGATCTGC	5′CGCTACACAAGTCGTAGTCTGG-3′
BECLIN	5′GGCTGAGAGACTGGATCAGG	5′CTGCGTCTGGGCATAACG-3′
PPIA	5′-AAATGCTGGACCCAACACAAA-3′	5′-TTGCCAAACACCACATGCTT-3′
